# Implementing nature-based solutions requires distinguishing place from space

**DOI:** 10.1007/s13280-026-02373-3

**Published:** 2026-03-26

**Authors:** Damon M. Hall, Charles B. van Rees

**Affiliations:** 1 Marine and Environmental Sciences, School of Public Policy and Urban Affairs, Northeastern University, Boston, MA USA; 2 430 Nahant Rd., 101 Edwards Bldg., Nahant, MA 01908 USA; 3 Odum School of Ecology, University of Georgia, 140 E. Green St., Athens, GA 30602 USA

**Keywords:** Biodiversity, Engineering with nature, Governance, Natural infrastructure, Nature-based solution, Sense of place

## Abstract

Nature-based solutions (NbS) scholarship is overshooting its most basic purpose—the implementation of NbS projects. Investment in high-level design is outpacing the practical realities of implementation. While expert-driven approaches are useful for project prioritization at landscape scales, all NbS projects are implemented in real places, inhabited by human and non-human lives. Projects span multiple properties, impact resident livelihoods, and affect and are affected by local and regional ecological dynamics. In the excitement of novel technical top-down NbS designs, equal attention is needed for ground-up social–ecological contexts of locales. Using geographers’ conceptual distinction between *space* and *place*, we outline a place-based inquiry useful for assessing site-level ecological and social feasibility to advance implementation. We identify implementation barriers, routes for top-down and bottom-up integration and call for case studies detailing implementation challenges and remedies. Integrating remote-spatial and place-based data enables contextualizing design—saving time and money and consequently increasing successful implementation.

## Introduction: Nature-based solutions as a new paradigm

To adapt to climate change while achieving the United Nations Sustainable Development Goals and addressing global challenges like water and food security and biodiversity loss, governments are increasingly turning to nature-based solutions (NbS). NbS are natural, artificial, or restored ecosystems that are managed, created, or protected to provide human well-being, ecosystem services, resilience, and biodiversity benefits (Nesshöver et al. 2017; IUCN 2022). NbS offer the special appeal of delivering these benefits in a climate-resilient manner while potentially providing jobs, economic innovations, and habitat for biodiversity. The win–win appeal of co-benefits has led to widespread interest from government agencies, conservation organizations, and sustainable development NGOs, with some labeling NbS as a *paradigm shift* denoting a radical shift in knowledge and thinking (Seddon et al. 2019; Bark et al. 2021; European Commission 2021; Xie et al. 2022). According to Kuhn (1962), a paradigm shift alters the questions science must address, forcing new standards of scrutiny, theoretical models, cases to firm up those models, and emergent problems to articulate. Thus, a marquis characteristic is an open-endedness that “leaves all sorts of problems for the redefined group of practitioners to resolve” (Kuhn 1962 p. 11). For NbS, the most significant unresolved problem is implementation—getting projects from a conceptual plan to a functioning installation (Brears 2020; Seddon et al. 2020; Adams et al. 2024; Castellar et al. 2024; Fang et al. 2024). Despite the dramatic acceleration of published literature on NbS demonstrating rapid interdisciplinary integration of necessary scholarly expertise, such advances have left a yawning absence of equal accelerations of successful implementation (Martin et al. 2025).

All NbS are localized interventions; that is, they must be implemented or constructed in a given location in geographical space (Kinol et al. 2023). In the process, they affect specific habitats, properties, communities (ecological and social), livelihoods, tax bases, biogeochemical processes, and ways of life within that locale. Advancing beyond theory and frameworks toward implementation requires understanding the particular ecological, political, economic, and social contexts of a site (Kumar et al. 2020; Pätzke et al. 2024) and how they might affect project performance, results, and longevity (Ibrahim et al. 2025). While top-down analyses for mapping and spatial prioritization are becoming a mainstay of NbS scholarship (Ronchi and Salata 2022; Cong et al. 2023; UNEP-WCMC 2023), from the use of satellite imagery to identifying NbS approaches that fit physical landscapes (Vera-Puerto et al. 2020; King et al. 2022; Moreau et al. 2022; Zhu et al. 2024), these approaches outweigh the logistically focused, place-based research and knowledge necessary for carrying out projects and closing the implementation gap. As a result, the practicalities of transitioning NbS concepts and principles into operational projects are underdeveloped, neglecting the inherently place-specific, context-driven functionalities of NbS (Albert et al. 2021) and contributing to the slow uptake and limited public acceptance highlighted as a major challenge by experts in the field (Cohen-Shacham et al. 2019; Fang et al. 2024; Martin et al. 2025).

Implementation of NbS projects is riddled with social, economic, and political barriers (Christopher et al. 2024; Solheim et al. 2021; Martin et al. 2025), unexpected ecological outcomes including nuisance species and compromised biodiversity benefits (van Rees et al. 2023a, 2023b; Brancalion et al. 2025), influenced by powerful external interests (Zhu et al. 2024), social inequities (Cook et al. 2025), and mismatches between project design and local context (McPhearson et al. 2025). Proceeding from academic scholarship to NbS implementation necessitates the less sexy clean-up work of a new paradigm—the effort necessary to “tidy up the space” that the new paradigm occupies (Kuhn 1962 p. 11). Most papers on NbS relegate discussion of implementation to the limitations section, characterizing implementation in terms of feasibility constraints (Zeng et al. 2020; Ellis et al. 2024; Knowles et al. 2025), political or social intractabilities (Filewood and McCarney 2023; Kinol et al. 2023), risks of ecosystem disservices or ecological traps (Zhou et al. 2024; Mohan et al. 2025), socioeconomic factors (Kim et al. 2024; Jiang et al. 2025), or the particularities of context (Dunlop et al. 2024). Where implementation receives attention, approaches have been criticized for limited one-size-fits-all approaches (Colléony and Shwartz 2019; Albert et al. 2021) and tokenistic local community engagement (Kiss et al. 2022; Ibrahim et al. 2025). What remains is little practical or conceptual guidance for addressing local barriers and, thus, actualizing appropriate and effective NbS projects. The gap widens.

To conceptualize overcoming the implementation gap, the theoretical distinction between *space* and *place* can elucidate top-down–bottom-up dialectics that are imperative to mainstreaming NbS. Scientific generalization advances NbS physical design principles, but implementing NbS projects requires particularization of context if NbS advocates are to overcome administrative, financial, ecological, and social-acceptance barriers.

## Top-down and bottom-up approaches

Implementation difficulties reflect familiar challenges of integrating top-down and bottom-up approaches and data. NbS achieve greatest benefit at the landscape scale (EU 2021), yet operationalizing projects occurs at local scales. The opposing concepts of *place* and *space*, as used in the field of geography (sensu Tuan 1990), are helpful in differentiating the considerations and tasks of interdisciplinary synthesis, framework development, and spatial prioritization on one hand, and the manifold logistical realities of local implementation in a given social–ecological system on the other. Dominant 20th-century trends in geography employed abstract spatial analyses in pursuit of universal theories about how physical geomorphology (space) drives human behavior (Entrikin 1991). Scholars formulated the concept of place^1^ to acknowledge the human nuances of how people live in and shape landscapes (Jackson 1984; Tuan 1990). Place offers empirical accounts of the affective and socio-cultural importance people ascribe to locales—such as how a particular house is considered a home by a person (Berdoulay 1989). An assessment of place involves understanding the particularities of what is meaningful to those who inhabit and shape that place (Cosgrove 1998). Shared meanings of a place influence behavior and intra-community dynamics (Williams and Vaske 2003; Brown and Raymond 2007), affecting support for or resistance to (e.g., not-in-my-backyard [NIMBY] politics) sustainable development like NbS (Hall et al. 2013; Devine-Wright 2022). Place captures a more complete social–ecological image of a space’s physical and ecological realities, as well as the socio-political context of imbued meanings, identities, and attachments humans give to that space.

**Fig. 1:**
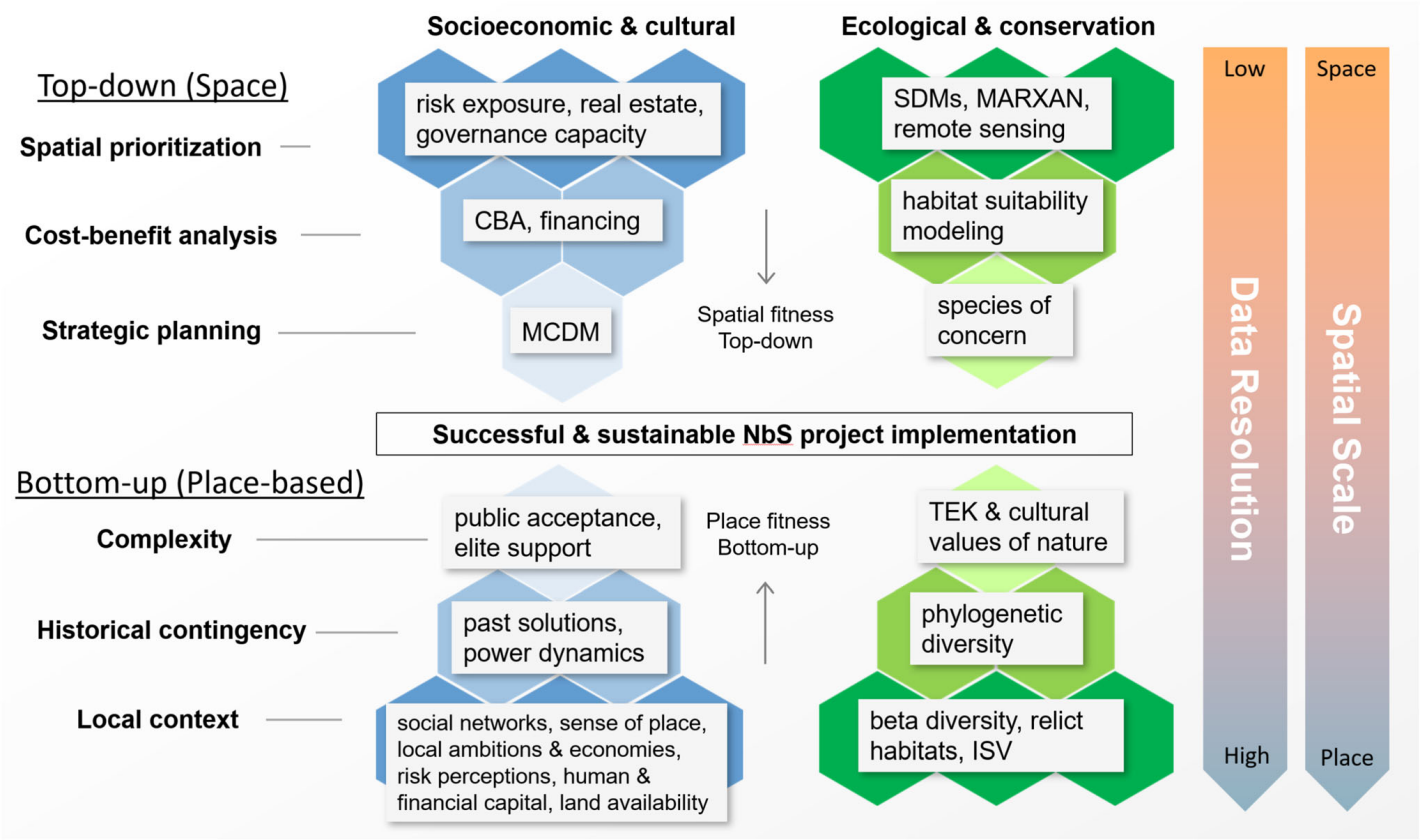
Comparison of top-down (space-focused) approaches and bottom-up (place-based) approaches to NbS design and implementation. CBA is cost–benefit analysis, MCDM is multiple criteria decision-making, SDM is species distribution model, MARXAN is software that aids in spatial prioritization for protected areas for biodiversity conservation, TEK is traditional ecological knowledge, and intraspecies variation, a fine-scale measure of biodiversity not captured by many top-down assessments

For NbS, researchers can identify the right *space* for a project hydrologically, geomorphologically, fiscally, and in terms of technical feasibility, but if it is the wrong *place,* socio-cultural and ecological nuances may impede successful implementation before or after a project has broken ground, with implications for long-term performance. Factors like land-tenure system, real estate dynamics, legacies of property ownership, community acceptance, political power dynamics, and interagency cooperation can stymie project viability and sustainability (Dorst et al. 2022; Chambers et al. 2023; Castellar et al. 2024; Christopher et al. 2024). Nuanced barriers are unique to a place and are, thus, rarely generalized across sites, yet understanding a place’s sociopolitical system, its history, and its people is critical for implementation. For NbS design and implementation, any assessment of *spatial fitness*—the conventional top-down approach using support tools like remote sensing and species distribution maps—must necessarily be complemented with an assessment of *place fitness*—a bottom-up understanding of local context and how it will respond to an NbS (Fig. 1).

Ecological factors in conservation planning and sustainable development are also conventionally viewed using a space-based or top-down lens (e.g., via species distribution models, remote sensing data, or upscaled climate models) yet observed landscape-scale patterns of biodiversity are the products of local ecological processes (e.g., individual survival and dispersal) and historical contingency (e.g., community assembly, geological history, extinction events). Biodiversity conservation science inherently involves the interaction of social factors with ecological management (Wickham et al. 2022). As NbS are increasingly valued for their potential biodiversity conservation outcomes (Pettorelli et al. 2021; van Rees et al. 2023a, 2023b, 2024), considering local ecological context, social dynamics, and the implications of their interactions co-equally will be of increasing importance (Maller 2021). When solely using generalized and top-down prioritization approaches, decision-makers risk missing important factors like rare species, beta diversity (Socolar et al. 2016; Kamran et al. 2020), local genetic or other intraspecies variation, and local environmental knowledge (e.g., Indigenous or traditional ecological knowledge; Nelson and Reed 2025). Furthermore, conventional top-down approaches to biodiversity conservation have been rightly criticized for being overly technocratic and contributing to ongoing environmental justice and social equity issues affecting Indigenous Peoples and local communities (IPLC) (Dowie 2011; Martin et al. 2016). For example, protected areas for biodiversity, guided mainly by large-scale spatial prioritization exercises using remotely sensed data and species range maps, are often designated by coalitions or governments with little support or input from local communities (Tauli-Corpuz et al. 2020). Mainstreaming NbS implementation provides an unprecedented opportunity to adopt a place-based paradigm of ecological restoration and environmental management that is better aligned with local governance structures and diverse values toward nature (Table 1; Turnbull et al. 2023).

**Table 1:** Comparison and complementarity of space-based and place-based approaches to nature-based solutions (NbS) implementation

Space-Based (Conventional) NbS Implementation Approaches	Place-Based NbS Implementation Approaches
Top-down	Bottom-up
Emphasis on present and future conditions	Emphasis on past and present, legacy, and carry-on effects
Defined by modeling and expert opinion	Defined by local knowledge and engagement
Prioritizing Alpha biodiversity	Prioritizing Beta biodiversity and endemism
Overlaying species distribution models	Collecting occurrence and detection data
Technical and academic process	Practical, implementation focus
Generalized	Contextualized and relationship-based
National and regional economic values	Local economic value, non-monetized socio-cultural values, reciprocity

## Socio-political barriers to nature-based solution implementation

Social-system barriers are routinely identified as the most obstructive to implementing NbS (Christopher et al. 2024; Solheim et al. 2021). There are three common barriers: bureaucrats’ familiarity with NbS approaches, local communities’ acceptance of projects, and acquisition of capital resources to finance projects.

### Knowledge barriers

The most frequently cited barrier to NbS implementation is a lack of knowledge about NbS by administrators and communities (Kabisch et al. 2016; Kumar et al. 2020; Megyesi et al. 2024; Martin et al. 2025). Ignorance of NbS design options, co-benefits, and feasibility of NbS construction and maintenance results in the omission of NbS from consideration (Abera et al. 2025). This unfamiliarity includes uncertainty about the delivery of co-benefits to mitigate risks (Solheim et al. 2021) and a fear of unfamiliar approaches (Scyphers et al. 2020; Kabisch et al. 2022; Cook et al. 2025). These uncertainties and fears are reflected in regulatory and institutional barriers (Serra-Llobet et al. 2022; Dunlap et al. 2024).

### Acceptance barriers

The second-most identified barrier to NbS implementation is a lack of social acceptance of NbS by administrators and communities (Ferreira et al. 2020; Anderson et al. 2021). Despite understanding what a local NbS design is and does, some communities decide they do not want it in their place for a variety of reasons. These may be because of costs or disproportionate burdens of projects (Opperman et al. 2009; Wijsman and Berbés-Blázquez 2022). Outcomes may clash with actors’ other values or administrative priorities, resulting in resistance to NbS (Pätzke et al. 2024). Where litigation can hamstring a project, local social acceptance is vital to successful NbS implementation (Anderson et al. 2021). Acceptance is influenced by many factors, including how well a project addresses a community’s agreed-upon problems, matches key stakeholders’ values and community ambitions, and adheres to timelines and budgets (Otto et al. 2023; Kühn and Vasstrøm 2024).

### Financial barriers

A third class of implementation barriers involves financial and property acquisition obstacles. NbS projects must assemble funds from various sources to finance projects. Some locations lack precedents for NbS projects. An absence of available public funding (Zeng et al. 2020; Toxopeus and Friedemann 2021) requires assembling a mixture of funding sources (Charry et al. 2022). Budget cuts and changes in private investors put projects at risk. Funders’ divergent interests, timelines, and requirements (e.g., cost-share, monitoring, benefit–cost ratios) pose additional challenges that shape a project (van Loon-Steensma and Vellinga 2014; Balmford et al. 2023; Kim and Kim 2024). These funding challenges lead some bureaucrats and administrators to view NbS projects and maintenance as prohibitively expensive (Wang et al. 2024) 

In projects where property acquisitions are necessary, the most substantial barriers are acquiring properties in different land-tenure contexts where space may be scarce and arranging relocations to implement a project can be difficult (Lawrence et al. 2020; Dorst et al. 2022). In rural areas, property owners may be unwilling to move or sell land at what administrators assess as a fair-market price (Solheim et al. 2021). In informal land-tenure systems, such as customary or traditional arrangements, land acquisition can unjustly displace vulnerable indigenous people and local communities (IPLC; Del Pino and Marquez 2023; Tallent and Zabala 2024). Real estate acquisition can disrupt budgets and timelines, entirely jeopardizing or reshaping the contours of a project.

## Overcoming implementation barriers with place-based data

Because the barriers between NbS design and implementation are contingent upon context, the types of information needed for executing NbS projects must be contextualized. Scholars describe this scale of inquiry with terms juxtaposed from top-down lenses including place-based (Cong et al. 2023; Perschke et al. 2023; Ibrahim et al. 2025; Pellery and Moghadam 2025), place-specific (Kabish et al. 2022; Han et al. 2023; van der Jagt et al. 2023), embedded in context (Serra-Llobet et al. 2022), context-specific (Solheim et al. 2021), customized, personalized (Wang et al. 2024), or contextualized (Seigerman et al. 2023). Researchers argue for gathering site-specific socio-cultural and political information, including knowledge of a community’s (Tafel et al. 2022; Pätzke et al. 2024) and administrator’s (Santoro et al. 2019) values, priorities, and attitudes to understand how projects may align or conflict with local needs, identities, and ambitions (Kumar et al. 2020). Methodologically, research design must be tailor-made (Hölscher et al. 2024) to the setting and conducted in a way that, at minimum, does no harm to working relationships among actors and, preferably, improves relationships. Therefore, gathering place-based data for NbS implementation requires (1) understanding and documenting local interests, politics, and power dynamics of stakeholders, (2) engaging interpersonally with community leaders in the project location, (3) detailing distributions of benefits and burdens, and (4) registering the historical context.

### Documenting local interests and power and governance dynamics

Where NbS projects use public and private funds to distribute advantages and burdens, implementation is a socio-political matter that includes actors of substantial power differences (Clark and Harley 2020; Kinol et al. 2023). Here, science and engineering are strategically selected by actors with competing interests to advocate for different NbS options (Thaler and Levin-Keitel 2016; Kiss et al. 2022). Understanding the specific contours of the political field—the elite actors, their ambitions and capacities, and the legal and administrative boundaries—can help those responsible for designing and implementing NbS facilitate successful projects.

International goals (e.g., UN Sustainable Development Goals, Conservation Biodiversity Framework), multinational incentive programs (e.g., Horizon Europe, European Union (EU) Green Deal, EU Biodiversity Strategy 2030, EU Adaptation Strategy), and national aims (e.g., United States’ Engineering with Nature, Costa Rica’s Campaign for Nature) provide an administrative structure for NbS implementation that can be analyzed from afar. However, an understanding of local context reveals key dynamics that influence administrative levers (Kemmis 1993; Honadle 1999; Rogers et al. 2023). For example, several nations require benefit–cost ratios to be > 1 to warrant public works funding. Calculations vary and are subject to analyst discretion concerning how much economic data to gather; therefore, actions at the local level both constrain and are constrained by the decisions of bureaucrats. Where benefit–cost ratios are binding, low-income and rural project locations tend to underperform; without adjustment, they are passed over (Frank 2000). Actors may pressure bureaucrats to find alternative routes to authorize projects and appropriate funds (Seipp et al. 2023). Where benefit–cost ratios are non-binding, bureaucrats play a significant role in bending decisions to bureaucratic preferences and local influence (Romsdahl et al. 2019; Hammes et al. 2021).

Regional governance is critical for aiding smaller communities (Selseng and Gjertsen 2024), yet each regional administrator differs in their priorities, risk perceptions, and attitudes toward community partners and NbS (Hammes 2021). A complexifying factor in resource-dependent communities is the embeddedness of agencies that increase the influence of elite local actors in NbS decision-making (Kretser et al. 2018; Huxham et al. 2023; Tallent and Zabala 2024). Place-based research uncovers these social networks of influence that explain barriers or facilitators of NbS implementation and other relevant site-specific influences such as election cycles and past efforts to address problems warranting NbS (Solheim et al. 2021).

### Engaging interpersonally with stakeholders

Many call for better community engagement in NbS projects (e.g., Seddon et al. 2021; van der Jagt et al. 2023; Kim and Kim 2024), advocating for the informational value, rather than any persuasive value, that engagement yields. Beyond fulfilling legal requirements or seeking “buy-in”, stakeholder and community engagement designed as research (Hall et al. 2016) can unearth a wealth of untapped IPLC knowledge, values, visions of the future, local political savvy, and historical perspectives (Hall et al. 2024a, 2024b). This information can help administrators overcome the inertia of a legacy of clumsy stakeholder and community engagement in engineering-based public works projects (Bryson et al. 2017) criticized for tokenistic and superficial participation intended to achieve legitimacy rather than to acknowledge the vital role communities play in advancing implementation (Puskás et al. 2021; Kiss et al. 2022; Fang et al. 2024). In contrast, authentic engagement is demonstrated by contributions and insights by communities to the NbS scoping, design, and implementation of a project (Wijsman and Berbés-Blázquez 2022). Calls for more “genuine” and “closer” engagement of communities reflect convergence around the need for place-based research for NbS implementation (Ferreira et al. 2020; Wamsler et al. 2020, p. 242; Kiss et al. 2022, p. 248). These calls include mandates for meeting with key stakeholders and communities in carefully planned communicative settings so they can speak candidly, have their voices documented and considered, and assume leadership roles (Diep et al. 2022; King et al. 2023).

### Documenting the distribution of benefits and burdens

NbS co-benefits and burdens are unevenly distributed across physical and social geographies, triggering support from those who stand to benefit and opposition from those who will bear costs (Kiss et al. 2022; Kim and Kim 2024). Burdens associated with NbS affect individuals, families, and organizations differentially. For families, these burdens may include relocating from home, selling the family farm, changing management practices and familiar behaviors, adopting a new livelihood, accepting a new future, coping with gentrification, losing a community, and accepting the uncertainty accompanying those choices (van Rees et al. 2024). For municipalities, burdens include abdicating tax revenue, spending down budgets on implementation costs, losing political capital, and changing economic infrastructure (Opperman et al. 2010; Watson et al. 2016). Anticipated benefits and burdens may be real or perceived, likely or unlikely. But when the continuity of livelihoods, land-uses, or access is threatened, local actors organize and deploy advocacy information and messaging, disrupt planning efforts, influence agendas, pressure agency staff and bureaucrats, target project funding sources, and advocate to their national and sub-national legislators (van Loon-Steensma and Vellinga 2014; Perlaviciute 2022; Hall 2024; Murunga et al. 2024). Cataloging the cascading co-benefits and burdens of an NbS project, its actors’ interests and positions (Pätzke et al. 2024), and the functioning of social networks (Mitincu et al. 2023; Tamasiga et al. 2024) reveals patterns of local acceptance and barriers to implementation. Those accepting the burdens must be accounted for and compensated. Left unaddressed, burdens may resurface as lawsuits or injustices.

Long-term residents are sources of valuable information about local social networks and structures. They know what ideas are likely to be favored or rejected by their neighbors and local opinion leaders. They know which families are willing and unwilling to sell land. They know who to talk with and who must be involved in decision-making for a new idea to be accepted locally as legitimate (Slavíková and Milman 2023).

Researchers should accept that local actors may mistrust government agencies and NGOs carrying out NbS (Davis et al. 2022). They may be skeptical of project funding sources. Key stakeholders and trusted stakeholder groups act as gatekeepers to the process or to community members, and elites in a community can shape decision-making by controlling flows of information. Particularly in small rural communities, outside experts must build interpersonal relationships and earn trust to be granted entry (Catalano et al. 2025). Outside project ideas must be made *local* (Diep et al. 2022; Kim and Kim 2024).

### Historical context

Anthropogenic landscapes are places shaped by a legacy of economic, cultural, and social choices that led to the formation of households, businesses, and institutions. Each place is weighted by history with an identity that is decidedly unique (Silverman 2017). Each contains unique assemblages of stakeholders nested within governance structures (Matson et al. 2016; Gober 2018). There is a cultural inertia to places that reinforces past choices and upholds current institutions and the social order (Lang et al. 2021). For example, project sites are often situated within a landscape of inequities that must be known and accounted for (Siegerman et al. 2023). Residents have local memories of past project successes and failures, winners and losers. Memories of interactions with governing and NGO organizations shape attitudes toward those agencies. In addition, the attributes of a place may be central in place attachment and identity, such that community acceptance of any project must include maintaining the beloved cobblestones, fishing spot, or ballfield. Omitting the importance of place attributes risks ending a project just as much as ignoring the historical social structure of that place.

In sum, the data needed for implementation must contextualize the distribution of risks, benefits, and burdens (Table 2). The aim is the particularization of barriers and facilitators to implementation in a project location. This assessment can aid decision-makers in improving NbS design to align local needs with the decision space to increase project satisfaction, local ownership, and local endorsement of NbS project design (Ferreira et al. 2020; Tamasiga et al. 2024).

**Table 2:** Summary data for a place-based understanding of major barriers to NbS implementation

Barrier	Site-specific project-specific details
Knowledge	Local familiarity with and perceptions of NbS as a concept and practice
Acceptance	Perceptions of the severity and distribution of costs, risks, and benefits Local attitudes toward the administering agencies and partnering organizations Influence of history: past efforts to address problems; places and events relevant to local identities Influence of issue severity: people are disproportionately benefited and harmed by the central problem and the NbS as a solution Influence of opinion leaders and identity politics
Financial	Status (and trajectory) of required land parcels, land ownership status—working land, probate, estate—and landowners’ visions for their lands

## Ecological and conservation considerations for place-based nature-based solutions

The ideal advanced in the literature is for NbS to facilitate regenerative relationships between humans and nature, where ecosystem services and biodiversity are supported via habitat restoration and creation, and where ecological processes and services are synergistic and self-reinforcing (Nelson et al. 2020; Welden et al. 2021). However, as with socioeconomic considerations, the necessary ecological knowledge to facilitate decision-making, account for and predict benefits, and strategically employ NbS is limited (van Rees et al. 2023a; Buckley et al. 2024; Frantzeskaki et al. 2025). Successful NbS implementation requires careful attention to their ecological structure, which is ultimately the driver of natural processes yielding co-benefits (i.e., ecosystem services) and the foundation for special characteristics of NbS that give them advantages over conventional approaches, for example resilience to change and regeneration from extreme events (Nelson et al. 2020; Vari et al. 2022). Furthermore, for NbS to fulfill promises of substantial benefits for nature conservation (i.e., biodiversity net-gain), careful attention to their ecological components will be necessary.

### Local-scale ecological factors for nature-based solutions

The ecological characteristics of NbS features and the biodiversity of surrounding ecosystems are critically important to their functioning and capacity to provide conservation co-benefits. While research on the ecology of NbS and evidence of their capacity to enhance biodiversity remains limited, the factors considered in NbS design and prioritization frameworks that include biodiversity reflect a top-down focus parallel to socio-cultural variables. This is especially reflected in the reliance on habitat metrics and estimates of species richness based on habitat associations characteristic of most biodiversity accounting and systematic conservation planning (Fletcher et al. 2025).

#### Species-level metrics

At the species level, considerable information is lost by focusing exclusively on taxonomic species richness (Fletcher et al. 2025). Qualitative information stemming from species identity (e.g., traits, protected status) is also particularly important (Noss 1990). For example, the establishment of invasive alien species may increase overall species richness in the short term, but these species may compromise ecosystem services or reduce native biodiversity in the long term. In addition, some species may be disproportionately sensitive to environmental conditions (umbrella species), functioning as a more useful metric of ecosystem condition. Still, other species may have greater economic or cultural importance to people (Meuser et al. 2009), or have certain legal protections, giving them context-specific importance.

Beta diversity is an important characteristic of biodiversity that may be missed in space-based analyses (Socolar et al. 2016). Beta diversity refers to the complementarity or “turnover” of biodiversity in one locale relative to another; in other words, its unique contributions to regional biodiversity (Whittaker 1960; Gaston and Williams 1996). Local ecological conditions, both present and historic, affect beta diversity and determine endemicity, which is an important component in conserving overall biodiversity (Fa and Funk 2007; Jost et al. 2010; Socolar et al. 2016). Endemic species are often missed by conventional national- or global-scale approaches, resulting in poor protection (Jenkins et al. 2015; Kraus et al. 2022; Gallagher et al. 2023). Beta diversity and endemism are especially important contributors to the overall biodiversity of artificial habitats and habitat patches in human-dominated landscapes (Gabriel et al. 2006; Clough et al. 2007), making these measures of special ecological concern in NbS implementation.

As in socioeconomic contexts, history is a critical part of the ecology and biodiversity of a place, although ecological history potentially involves much longer time scales. Phylogenetic diversity—the variety of evolutionary history captured by the assemblage of a given area (Faith 1992)—may well serve to capture and compare differences in evolutionary history that can be supported by a given NbS. Richness-based measures consider all species to be of equal value, but different species may carry very different amounts of evolutionary information and may be unique in their conservation importance (Vanewright et al. 1991; Faith 1992). Metrics like the Equal Splits Measure (Redding and Mooers 2006) that express the phylogenetic uniqueness and evolutionary information captured by different taxonomic groups in millions of evolutionary years better assess the place-based importance of local species and species assemblages (Cadotte and Davies 2010). Methods like these have been used successfully in place-based biodiversity analyses of human-dominated landscapes (Hill et al. 2019) and would be useful for place-based NbS implementation.

#### Intraspecies-level metrics

At a finer resolution, other forms of biological diversity manifest at local scales at the intraspecific level (Mockford et al. 2005; Kraus et al. 2022). Subspecies (and other local phenotypes or varieties) are locally aggregated in space and are therefore particular to specific locales (i.e., places), representing important contributions to local biodiversity (Des Roches et al. 2021). These subspecies may be specifically adapted to local conditions or simply distinct because of a history of isolation and long-term genetic drift, or founder effects, and they constitute a valuable, fine-grained component of biodiversity (Des Roches et al. 2018; Paz-Vinas et al. 2018). Local genetic variation is important to ecosystem function and services, making it an important component of place-based NbS planning (Des Roches et al. 2021). Small, isolated, and relict habitats in anthropogenic landscapes are increasingly recognized for their contributions to local biodiversity (Wintle et al. 2018), suggesting that NbS designed with place-based priorities might have considerable local-scale conservation value.

Intraspecies genetic variation, measured using genetic markers, is a more direct and potentially finer-scale metric capturing local biodiversity. While biodiversity declines are readily apparent in reduced census numbers and elevated species extinction rates, declines in intraspecific variation often occur more rapidly and over shorter time spans, with real consequences for ecosystem service delivery (Des Roches et al. 2021). Top-down analyses using pooled data neglect species-level details like life history, generation time, movement behavior, and mating systems, leading to potentially misleading assertions about the stability of intraspecific genetic variation (Millette et al. 2020; Paz-Vinas et al. 2021). Place-based, species-specific natural history knowledge is required to contextualize intraspecific conservation (Leigh et al. 2021) and should be included however possible in NbS design and implementation.

### Place-based biodiversity conservation in nature-based solutions implementation

The conventional prioritization frameworks applied to NbS implementation, as in the broader field of conservation science, are largely top-down in nature (e.g., Berg et al. 2024; Coleman et al. 2024), similarly risking the neglect of important fine-scale nuance and local context as for social variables, and with similar risks for compromising successful and long-lasting NbS implementation (Richards et al. 2024).

Top-down approaches include Systematic Conservation Planning, species distribution modeling, and protected area design, which are chiefly focused on habitat-suitability indices, measures of species richness, and remotely sensed environmental data, leveraged with limited amounts of *in situ* measurements of biodiversity (Gauthier et al. 2010; Kullberg et al. 2015). Legitimate concerns have been raised that conventional approaches miss important components of biodiversity at best and perpetuate environmental injustices at worst (Fletcher et al. 2025). These approaches may contribute to fortress conservation (Bray and Velazquez 2009; Tauli-Corpuz et al. 2020) or conservation imperialism, whereby IPLC in low-income countries are forced to bear the brunt of the opportunity costs of nature protection while being excluded from resources. The displacement and exclusion of IPLC and the implementation of contextually inappropriate conservation solutions cause social harm (Dahlberg et al. 2010; Bontempi et al. 2023), reinforcing the notion that conservation has been advancing science (top-down) over practice (bottom-up; Schultz 2011).

Expert knowledge and cutting-edge modeling are certainly critical to designing and implementing NbS projects; however, relying only on technocratic solutions to the exclusion of local communities, values, and epistemologies is limiting (Daily and Matson 2008; Spangenberg et al. 2015; Berthet et al. 2022; Nelson and Reed 2025). Ignoring valuable socio-historical place-based knowledge results in oversimplifications of ecological complexity and poor accounting of potential tradeoffs between ecosystem services (Kronenberg and Hubacek 2016). This issue is especially prominent in freshwater ecosystems, which deliver a multitude of extremely valuable ecosystem services and support the most endangered portion of Earth’s biota, but which require careful place-based conservation solutions (Gorenflo and Warner 2016; van Rees et al. 2021; Vollmer and Harrison 2021; Vollmer et al. 2023). Further, decision-making for NbS and related ecological restoration will be increasingly driven by artificial intelligence (AI) due to burgeoning interest and support from funding institutions (Richards et al. 2024; Prodanovic et al. 2024). Growing reliance on AI-based methods, in conjunction with reduced support for *in situ* biodiversity data collection and a potential devaluation of local ecological knowledge may increase the risk of potential harms to local communities through inappropriate action or unethical use of their knowledge and information (Pritchard et al. 2022; Parris-Piper et al. 2023).

Emerging conservation approaches called for by the Kunming-Montreal post-2020 Global Biodiversity Framework (CBD 2022) emphasize the integration of local biodiversity governance with NbS through local biodiversity strategy and action plans (known as LBSAPs), placing greater priority on capturing local ecological dynamics (Xie and Bulkeley 2020) and taking a step toward the kind of place-based approach needed for successful NbS. As with any other institutional action, conservation interventions affect IPLC and benefit from increased social sustainability when co-produced with local involvement (Waylen et al. 2010). This is especially true in the context of NbS, wherein benefits to local people and ecosystems are the primary goals (Seigerman et al. 2023; Woroniecki et al. 2023). However, guidance and frameworks to usefully integrate just conservation measures with NbS implementation remain at early stages of development (McKay et al. 2023; van Rees et al. 2023b; 2024; 2026). The conservation science literature offers diverse conceptual framings and lessons learned to enhance a place-based approach to biodiversity conservation in NbS that might complement conventional approaches.

#### Place-based biodiversity conservation

Place-based thinking has been a part of the conservation biology literature since the 1990s (Norton and Hannon 1997; Williams et al. 2013) and is worth leveraging in NbS planning and implementation. Place-based conservation practice is tailored to the ecological and cultural idiosyncrasies of an area (Mason 2007) and designed to achieve conservation interventions that are socioeconomically sustainable in the context of a given social-ecological system. Frameworks for place-based ecological restoration and management (Wickham et al. 2022; Berthet et al. 2022) emphasize how exclusively top-down approaches to NbS can result in scale mismatches around the equitable delivery and maintenance of ecosystem services and the biodiversity that produces them (Cumming et al. 2006; Berthet et al. 2022). These approaches emphasize community engagement, collective action, and co-production with IPLC in concert with expert-driven information and decision-making (Büscher and Fletcher 2019; Berthet et al. 2022). Implementing such approaches necessitates specific norms of conduct and behavior (Wickham et al. 2022) and is intended to make conventional practices compatible with local belief systems and attachment to place while also catering to the ecological context (e.g., species of concern, dominant environmental threats). Importantly, place-based conservation efforts recognize the role of local communities in shaping and stewarding the biodiversity of the local human and natural landscape, a historically neglected factor that has affected the selection of restoration and conservation targets in the past (Hallett et al. 2013).

From the standpoint of biodiversity conservation, the NbS paradigm shift is an unprecedented opportunity for large-scale ecosystem restoration, with major implications for achieving conservation targets (CBD COP 2022; Key et al. 2022; van Rees et al. 2023a, 2023b; 2024). Since many forms of NbS implementation will involve planting structurally important vegetation like canopy trees or marsh grasses (Suedel et al. 2022; Chambers et al. 2023; Coleman et al. 2024), place-based conservation practice can also contribute to ensuring the compatibility of planting stock with local ecosystems. Propagules taken from local stock can help capture and preserve local intraspecies genetic diversity and increase the likelihood of viability through interactions with local mutualisms (e.g., mycorrhizal fungi) and consumers (e.g., lepidopteran larvae).

#### Indigenous and local knowledge and environmental justice

It is critically important to recognize IPLC not only as vulnerable to potential harms from conventional, market-driven, and top-down approaches to NbS, but also as essential collaborators and valuable contributors to successful local implementation. Biodiversity monitoring is critical to understanding the conservation value of existing and planned NbS (van Rees et al. 2022, 2023a, 2023b; Coleman et al. 2024). The contributions of local and Indigenous knowledge and community participation in biodiversity inventories and monitoring are increasingly appreciated in ecology (Luzar et al. 2021) and contribute important information that might otherwise not be captured. Local communities in general can play a large role in generating cost-effective, crowd-sourced biodiversity monitoring data in their locale (Danielsen et al. 2005; Kuhl et al. 2020). The advent of biodiversity-centric mobile apps for reporting and managing data greatly enhances the potential contributions of IPLC to biodiversity monitoring of local NbS (Howard et al. 2022).

The notion of braiding Indigenous and scientific knowledge on local ecology and biodiversity (Reed et al. 2022) is critically important for place-based NbS implementation, leveraging both an expert understanding of ecosystem functions in general and local understanding of history and nuance endemic to an area (Nelson and Reed 2025). Accordingly, approaches like “two-eyed seeing” (Reid et al. 2021) should be embraced wherever possible to capture different values and understandings of biodiversity and promote a more socio-ecologically sustainable perspective of reciprocity and co-existence with non-human life (Maller 2021; Welden et al. 2021). In co-producing a more complete picture of local ecosystems and their biota, experts and NbS planners must be mindful of the data sovereignty of IPLC, respecting proper research ethics, preventing biopiracy, and protecting ancestral rights to land and resources (Cottrell 2022).

## Applying place-scale knowledge to NbS implementation

New paradigms require a lot of “mop-up work” (Kuhn 1962 p. 11). For NbS, there is much to do to align the practices of design to implementation. The NbS paradigm shift has thrown the doors open to new ways of combining engineering, ecology, and sustainable development. Each perspective has its strengths, its good and bad habits, as well as its blind spots or trained incapacities—where one’s abilities and disciplinary specialties function equally as a way of seeing and a way of not seeing (Burke 1984). Accordingly, both prevailing practice and logistical ease drive the overdominance of top-down information and procedures in emerging NbS implementation. In both ecological and social disciplines, most available data are space based (i.e., detached from local context, scrubbed for generalizability) rather than place based. Place-based data are more time-consuming to obtain—for example, in situ biodiversity surveys or interviews with stakeholders—and can be difficult to distill down to formats that fit easily in large databases. Metrics that reduce this complexity facilitate broader comparisons, but reducing the resolution of data used to develop NbS poses the risk of losing important local information that can advance implementation.

Each NbS project site has become a candidate for NbS because its natural and built landscapes no longer fit its changing climate. Place-based approaches to NbS can illuminate differences in the potential for implementation success between prospective sites. For example, place-based data advance the salience of a project’s design by using local context to evaluate logistical feasibility (Clark et al. 2016). Contextual attributes can complement large-scale site suitability assessments for comparing candidate sites in terms of implementation feasibility. As an example, candidate sites X and Y have been assessed as equally effective NbS projects in terms of their physical and ecological conditions, species distribution potentials, and modeled restoration trajectories. Site X has a pair of local influential leaders in support of the project and 3 of 5 property owners willing to sell needed land. Site Y has no local leaders in support, a history of mistrust with the administering agency, and only 2 of 7 property owners willing to discuss selling land. These local-scale sociopolitical data indicate that investment in technical assessment of site X is more efficient and prudent than of site Y. When financial resources are limited, comparing sociopolitical feasibility can aid decisions about where best to invest full-scale technical feasibility studies. This type of integration of top-down and bottom-up assessments is vital to implementation; therefore, achieving parity between these viewpoints is crucial.

We call for more (1) published case studies focusing on attributes of implementation challenges, remedies used, and (2) the ways that place-based data (ecological and social) are integrated, or not, with space-focused data throughout NbS planning and implementation. Cases would contribute to understandings of NbS acceptance by agencies and local communities, as well as the proliferation and success of on-the-ground implementation. (3) Negative case studies—cases of unsuccessful top-down–bottom-up integrations—examining reasons for failed implementation with attention to place-based dynamics, are also needed. We also call for (4) more thorough articulation of place-based methodologies for reproducibility and theory building. Finally, (5) scholars and practitioners experimenting with place-based methodologies should articulate where adjustments to technical, community engagement, and monitoring decision-making were altered due to place-based findings. The place–space distinction should make communicating the data types and integration decisions easier.

Nature-based solutions exemplify a turning *toward* transdisciplinary practice (Suedel and Oen 2021; Frantzeskaki et al. 2025); its strength is its integrative and systemic approach to addressing societal challenges (Nesshöver et al. 2017). New paradigms equally mark a turning *away from* old ways of thinking. Inherited NbS design practices that privilege distanced study, experts’ voices over local, and researchers’ sampling convenience over site-scale data perpetuate implementation woes. Integration is needed. Place-based approaches to NbS promise improved feasibility and longevity because they fit the contours of a setting’s local ecosystem functions and human behaviors that created and perpetuate the very problems that originated the need for NbS. Spatial approaches to site selection, climate and species modeling, and overall project impact are necessary to initiate NbS projects, but a complementary suite of place-based data is also needed to ground-truth top-down lenses, to contextualize projects within their local sociopolitical–ecological landscapes, and to obtain community buy-in, which is critical for NbS project success. If societies at a global scale are to realize the climate-change-fighting potential of NbS with any urgency, it will be essential to complement space-based physical feature suitability assessments with place-based contextualization at the local scale.

## Data Availability

We do not analyze or generate any datasets because this work proceeds from peer-reviewed literature. All sources used are in the References section.

## Footnotes

1 Our use of place here should not be confused with lay accounts of place as an alternative expression of “community” (Wearne and Riedy 2024).
